# IgGs from Human Milk Hydrolyze microRNAs

**DOI:** 10.3390/molecules25102366

**Published:** 2020-05-20

**Authors:** Ivan Yu. Kompaneets, Evgeny A. Ermakov, Sergey E. Sedykh, Valentina N. Buneva, Georgy A. Nevinsky

**Affiliations:** Institute of Chemical Biology and Fundamental Medicine, SD of Russian Academy of Sciences, 8 Lavrentiev Ave., 630090 Novosibirsk, Russia; kompaneecivan@mail.ru (I.Y.K.); evgeny_ermakov@mail.ru (E.A.E.); sirozha@gmail.com (S.E.S.); buneva@niboch.nsc.ru (V.N.B.)

**Keywords:** Human milk, antibodies, Abzymes, miRNAs hydrolysis

## Abstract

Mother’s milk provides breast-fed infants with various nutrients, including peptides, proteins, DNA, RNA, antibodies, and other bioactive components promoting neonatal growth and protecting infants from viral and bacterial infection. The functions of many human milk components regarding the nutrition and protection of newborns may be very different compared to those of various biological fluids of healthy adults. For example, human milk contains catalytic antibodies (abzymes) with protein, lipid, and oligosaccharide kinase activities, which are absent in the biological fluids of healthy people and autoimmune patients. Obviously, the nutrition of infants with fresh breast milk is a special phenomenon having a very specific and important role. Here, we have shown that mother’s milk IgGs effectively split homo-(pN)23, and four miRNAs: miR-137, miR-219a-5p, miR-219-2-3p, and miR-9-5p. It was shown that ribonuclease activity is a unique property of milk IgGs. On average, individual IgGs hydrolyze (pA)23, (pU)23, and (pC)23 nonspecifically and with comparable efficiency, whereas the hydrolysis of four miRNAs is predominately site-specific. The specific sites of the hydrolysis of four miRNAs by IgGs from the blood of schizophrenic (SCZ) patients and secretory immunoglobulins A (sIgAs) from human milk were found earlier. The sites of the hydrolysis of four miRNAs by milk IgGs and sIgA-abzymes are almost the same, but are significantly different in comparison with those for SCZ IgGs. In addition, in contrast to the SCZ IgGs, milk IgGs and sIgAs efficiently hydrolyzed miRNAs in the duplex regions formed by their terminal sequences.

## 1. Introduction

Mammalian milk contains various components: RNAs, DNAs, antibodies (Abs), proteins, peptides, etc. [1,2,3]. Many compounds of breast milk are essential for neonatal growth and protect neonates from infections; they are usually regarded as integral parts of the intestinal physiology of infants [1,2]. Milk is a very important source of various proteins; more than 200 publications describing the milk proteome have appeared in PubMed over 17 last year [2], including NMR-based metabolic analyses providing a rapid characterization of the composition of breast milk [3].

Antibodies (Abs) to stable analogs of chemical reaction transition states and natural immunoglobulins (Ig) of living organisms with catalytic activities are called abzymes (ABZs) and are described in the literature [4,5,6,7,8,9,10]. The blood of patients with autoimmune diseases (AIDs) usually contains various auto-Abs-abzymes hydrolyzing many different compounds. They can be Igs directly to antigens, mimicking the transition states of chemical reactions or secondary anti-idiotypic antibodies-abzymes against active centers of enzymes ([4,5,6,7,8,9] and references therein).

The existence of ABZs in blood is a clear, statistically significant sign of the development of autoimmune processes [4,5,6,7,8,9,10]. To date, IgGs and/or IgAs, IgMs hydrolyzing, DNA, RNA [10,11,12,13,14], polysaccharides [15,16,17,18], oligopeptides, and proteins [19,20,21,22,23,24,25,26] were found in the blood of patients with various AIDs and some viral diseases (for review see ([4,5,6,7,8,9,10]). There are no any abzymes in the blood of healthy donors [4,5,6,7,8,9,10].

Human milk contains IgGs (5–7%), IgMs (<4–7%), IgAs, and secretory immunoglobulin A (sIgAs), of which sIgAs are the major ones (>85–90%) [27,28]. During the first 5–6 months of a newborn’s life, their immune system is not operational, i.e., the mucous surfaces of different tracts contain no Abs [28]. Newborns begin to produce Abs in the intestine only in the first 3–6 months of life. To a large extent, newborns are protected by Abs against bacterial, viral, and other harmful compounds from the mother’s milk (passive immunity), which fill up the baby’s mucous membranes [28]. In addition, small subfractions of milk IgGs and sIgAs hydrolyze DNA, RNA [29,30], NMPs, NDPs, and NTPs nucleotides [31], polysaccharides [32], proteins [33], and possess phosphatase activity [31].

During pregnancy and immediately after the beginning of the lactation, women demonstrate a sharp increase of autoimmune (AI) reactions similar to those in patients with AIDs, such as SLE, antiphospholipid syndrome, MS, renal insufficiency, Hashimoto thyroiditis, etc. [34,35,36,37,38]. Pregnant females may be immunized by different compounds of food, bacterial, or viral origin, which can stimulate the synthesis of various Abs and ABZs [5,6,7,8,9,29,30]. Women’s blood during pregnancy and lactation contains DNA similar to the blood of patients with several typical AIDs [5,6,7,8,9] and fetal cells [39]. During pregnancy and soon after childbirth, several AI pathologies can be “activated” or “triggered” in healthy women [29,30,31,34,35,36,37,38,40]. The beginning of lactation may be considered as an important period associated with the production not only of different auto-Abs, but also of very specific milk abzymes ([5,6,7,8,9] and references therein).

First, it was suggested that various AIDs are associated with defects of hematopoietic stem cells [41]. Later, it was proved that the development of AIDs is associated with significant changes in the differentiation profiles of bone marrow stem cells (BMSC) [42,43,44,45,46]. During the spontaneous development of deep systemic lupus erythematosus (SLE) in special SLE prone MRL-lpr/lpr mice [42,43,44] and experimental autoimmune encephalomyelitis (EAE) in EAE prone C57BL/6 mice (a model of human multiple sclerosis) [45,46], specific and similar reorganizations of immune systems occurred at the beginning of the production of abzymes. At first, these ABZs hydrolyze DNA and myelin basic (MBP) with low catalytic activities (conditionally prediseased mice). A significant increase in relative catalytic activities is associated with a transition from predisease to deep pathology, which correlates with additional changes in the differentiation profile of BMSC [42,43,44,45,46]. However, these processes are nearly the same in mice with AIDs and in healthy lactating mice [42,43,44,45,46]. These changes in mice that are healthy and lactating, and in mice with deep SLE pathologies, are accompanied by the production of ABZs with high catalytic activities. However, changes in the profile of differentiation of BMSCs in lactating mice are temporary and return to baseline three to six months after birth, while in mice with deep SLE, they are permanent [42,43,44].

Abzymes with low catalytic activities may be detected in women’s blood mainly in the third trimester of pregnancy [30]. The dynamic of increase in DNase ABZs concentrations correlates with the dynamics of changes in blood DNA concentrations and an increase in the level of cell apoptosis. For IgGs, the half-life was found to be ~15.7–29.7 days [47].

The peculiarity of the reorganization of pregnant women’s immune systems is associated with the “switching-on” of a special “immune-memory”, accumulating, during the third trimester of pregnancy, all information about environmental factors which may be dangerous for the child [5,6,7,8,9,42,43,44]. This information is partially “used” by the immune system of women during pregnancy, but is more effective immediately after the onset of the lactation. The immunization of females with different antigens 1–3 months (but not more) before the giving birth results in the appearance of different antigens in very high concentrations in milk Abs and ABZs [5,6,7,8,9]. The existence of abzymes in females’ blood and milk before and especially after childbirth may only be a part of the specific process of the restructuring of women’s immune systems.

Different abzymes of patients with several AIDs and Abs from the milk of lactating females catalyze the hydrolysis of various substrates. Natural abzymes catalyzing not only hydrolysis but also synthesis reactions were found only in human milk. Human milk IgGs and sIgAs phosphorylate more than 15 different milk proteins [48,49], unique lipids [50], and specific polysaccharides [51,52]. Overall, the immune system of lactating females is very specific and different from those of men and nonpregnant women. Milk is a unique source of different specific compounds, unusual Abs, and ABZs with both typical-for-AIDs and unique catalytic activities [4,5,6,7,8,9,48,49,50,51,52]. Therefore, the study of the functions of various components of fresh breast milk seems especially important.

Noncoding short microRNAs (miRNA) are both intra- and extra-cellular and are found in different body fluids of mammals [53,54]. They regulate up to several hundred genes and have many other, different biological functions [55,56]. Human milk contains many different cells which may be regulated by miRNAs. Various changes in miRNAs (miRNA-regulated gene network) can lead to very different cell alteration and the expression of many genes. Human milk can contain several tens to thousands of different miRNAs [56,57,58,59,60,61,62]. These are important for the functions of the lactating breast and for infants [57]. The data obtained strongly support the notion that milk miRNAs can enter the systemic circulation of the fed infant and provide tissue-specific immunoprotective and developmental functions [57]. 

Taking into account the important role of milk miRNAs in the proliferation, differentiation, and maturation of cells, it was important to analyze a possible synthesis of specific ABZs against any milk miRNAs. Currently, abzymes hydrolyzing miRNAs are found in the blood of patients with schizophrenia (SCZ) [63,64]. In addition, there were analyzed sIgA abzymes from female’s milk [65]. Here, a quantitative analysis of miRNA hydrolysis by IgGs isolated from fresh mother’s milk was performed. In addition, the substrate specificity of milk IgGs, sIgAs, and SCZ IgGs in the hydrolysis of four miRNAs was compared.

## 2. Results

### 2.1. Purification and Characterizing of IgGs

IgGs and IgAs from sera of healthy humans (except the milk of lactating women, and blood of pregnant women) could not hydrolyze DNAs and RNAs [4,5,6,7,8,9,10]. In this study, we analyzed the relative activities (RAs) of seven individual IgGs from milk samples of healthy lactating mothers in the hydrolysis of three different ribo-ONs ((pA)_23_, (pC)_23_, and (pU)_23_) and four miRNAs (miR-9-5p, miR-137, miR-219-2-3p, and miR-219a-5p).

**Figure 1:**
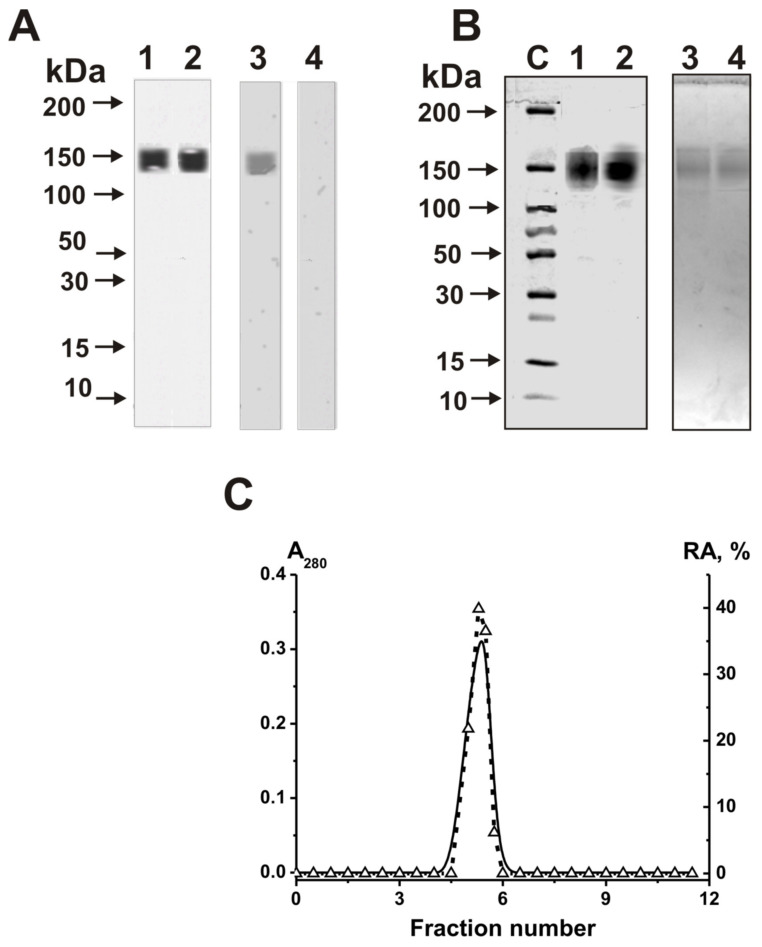
SDS-PAGE analysis of homogeneity of 12 µg IgG1 (lane 1) and IgG_mix_ corresponding to the mixture of seven individual IgGs (lane 2) in a nonreducing 3–18% gradient gel with the following staining by silver (**A**). IgG_mix_ preparation was analyzed by standard Western blotting (A). After SDS-PAGE, IgG_mix_ was transferred on nitrocellulose membrane. The membrane was treated with monoclonal mouse Abs (conjugated with horseradish peroxidase) against human IgGs (lane 3) or ribonuclease A (lane 4). Strict criteria verification was performed to prove that the ribonuclease activities of IgG1 and IgG_mix_ are their intrinsic properties. After SDS-PAGE of IgG1 (lane 1) and IgG_mix_ (lane 2) in a gel containing polymeric RNA, SDS was removed, and the gel was stained with ethidium bromide. The gel sections containing no RNA due to hydrolysis were not stained; dark bands on a uniformly fluorescent background, lanes 3 and 4 of Panel **B**. A part of the gel was stained with Coomassie R250 to show the position of intact IgGs (lanes 1 and 2) (**B**). Lane C shows the positions of protein molecular mass markers (B). Analysis of IgG_mix_ ribonuclease activity; (--) – A_280_ profile; (∆), relative activity (%) of IgG_mix_ in the hydrolysis of 5’-Flu-miR-137 after Abs FPLC gel filtration in acidic buffer (pH 2.6) (**C**). Complete hydrolysis of the substrate for 7 h was taken for 100%. The error in RAs from two experiments did not exceed 7–10%.

Electrophoretically homogeneous IgGs were purified from seven milk samples by affinity chromatography of milk plasma proteins, first on Protein G-Sepharose, and then by FPLC gel filtration in the buffer (pH 2.6), effectively destroying immune complexes, as described in [29,30,31,32,33]. The homogeneity of IgG samples was established using a mixture of equal amounts of polyclonal Abs from seven milk samples (IgG_mix_) (Figure 1A; silver staining). IgG_mix_ contained only typical IgGs of 150 kDa.

### 2.2. Application of the Strict Criteria

First, the IgG_mix_ preparation after SDS-PAGE and silver staining was electrophoretically homogeneous (Figure 1A, lanes 1 and 2). In addition, IgG_mix_ gave a positive immunoblotting response with mouse antibodies against human IgGs (Figure 1A, lane 3), but a negative response with mouse Abs against human ribonuclease A (Figure 1A, lane 4).

Moreover, to prove that ribonuclease activity belongs to electrophoretically homogeneous milk IgGs (Figure 1A) and not to copurifying classical RNases, we performed an in situ analysis of this activity. IgG_mix_ was subjected to SDS-PAGE (Figure 1B) using gels containing semipolymerized polymeric yeast RNA. After SDS-PAGE and the refolding of Abs, the gels were stained with ethidium bromide. The position of the dark band on the fluorescent background of the gels coincided with the positions of only intact IgG_mix_ (Figure 1B). Human canonical RNases have lower molecular masses (13–15 kDa) than intact IgGs (~150 kDa). SDS usually disassociates all stable protein complexes. Therefore, the absence of any protein bands except the IgG-band, and the lack of ethidium bromide staining in the gel zones of only intact IgG_mix_ (Figure 1B), provide direct evidence that IgG_mix_ hydrolyzes polymeric RNA. In addition, IgG_mix_ was subjected to FPLC gel-filtration on the column equilibrated with acidic buffer (pH 2.6), destroying strong immune complexes (Figure 1C). Peaks of IgG_mix_ and RNase activity completely coincided, and there were no other peaks of any proteins or activity.

In a large number of articles, it has previously been shown that the purification of IgGs on protein G-Sepharose followed by gel filtration in an acidic buffer (pH 2.6), destroying even stable immunocomplexes, yields antibodies that do not contain any enzymes [4,5,6,7,8,9]. In this paper, we confirmed the previously obtained data.

### 2.3. Hydrolysis of Homo-Oligonucleotides

**Figure 2:**
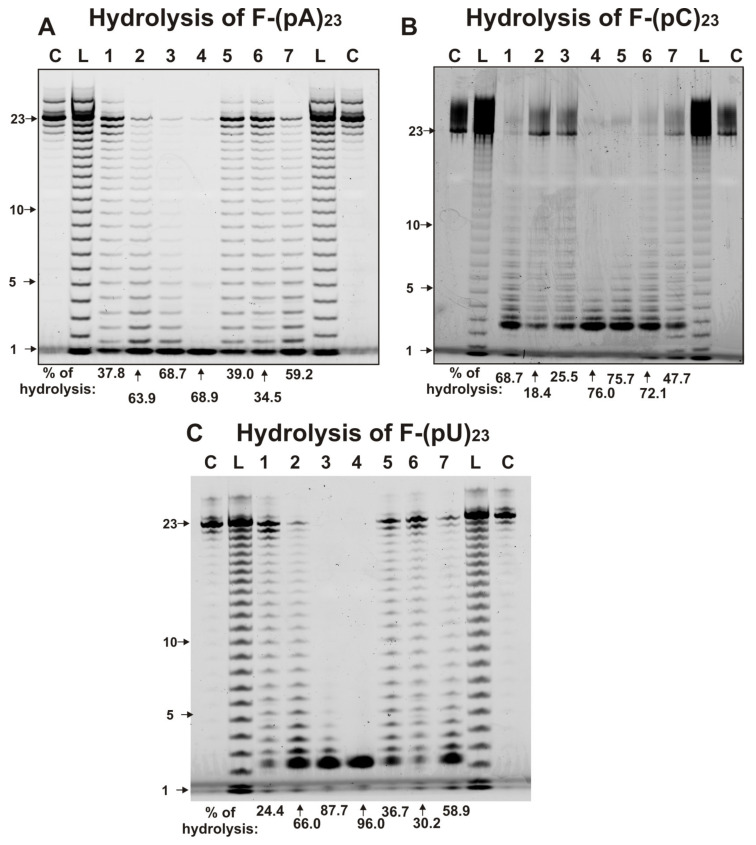
The patterns of F-ribo-ONs (0.01 mg/mL) splitting by seven individual milk IgGs (40 µg/mL). The products of the hydrolysis were revealed after mixture incubation for 1 h and subsequent 20% PAGE electrophoresis; the relative fluorescence (%) of intact substrates and the products of their hydrolysis was estimated. The numbers of IgGs, lengths of the products, and percentage of ribo-ONs hydrolysis by each IgG preparation are indicated in panels **A–C**. L lanes correspond to the markers of ONs lengths, while C lanes correspond to the substrates incubated without IgGs. The percentages of ONs hydrolysis correspond to the average values of three independent experiments.

To estimate the ribonuclease activity of individual IgGs, fluorescent (F) derivatives of ribo-ONs [(F-(pA)_23_, F-(pC)_23_,and F-(pU)_23_] and miRNAs [F-miR-137, F-miR-219a-5p, F-miR-9-5p, F-miR-219-2-3p] were used. Figure 2 demonstrates the typical patterns of ribo-ONs hydrolysis. The measured relative activities (RAs) of IgGs in the hydrolysis of three ribo-ONs in the same conditions were very different. Over 1 h, some IgGs nearly completely hydrolyzed all ribo-ONs (Figure 2). Taking into account the hydrolysis of ribo-ONs by some especially active IgGs for 10–20 min, it was concluded that all Abs samples hydrolyzed these three substrates almost nonspecifically, at practically all internucleoside bonds and with comparable efficacy.

The CC of the RAs between (pA)_23_ and (pU)_23_ was positive and high (+0.96), while it was were negative for (pA)_23_ and (pC)_23_ (-0.61) and for (pU)_23_ and (pC)_23_ (−0.42). It is interesting that the shortest major product of (pA)_23_ hydrolysis was a mononucleotide, while for (pC)_23_, and (pU)_23_ it was three nucleotides; however, in the latter cases, less effective formation of mononucleotides was also observed (Figure 2).

### 2.4. Hydrolysis of miRNAs

**Figure 3:**
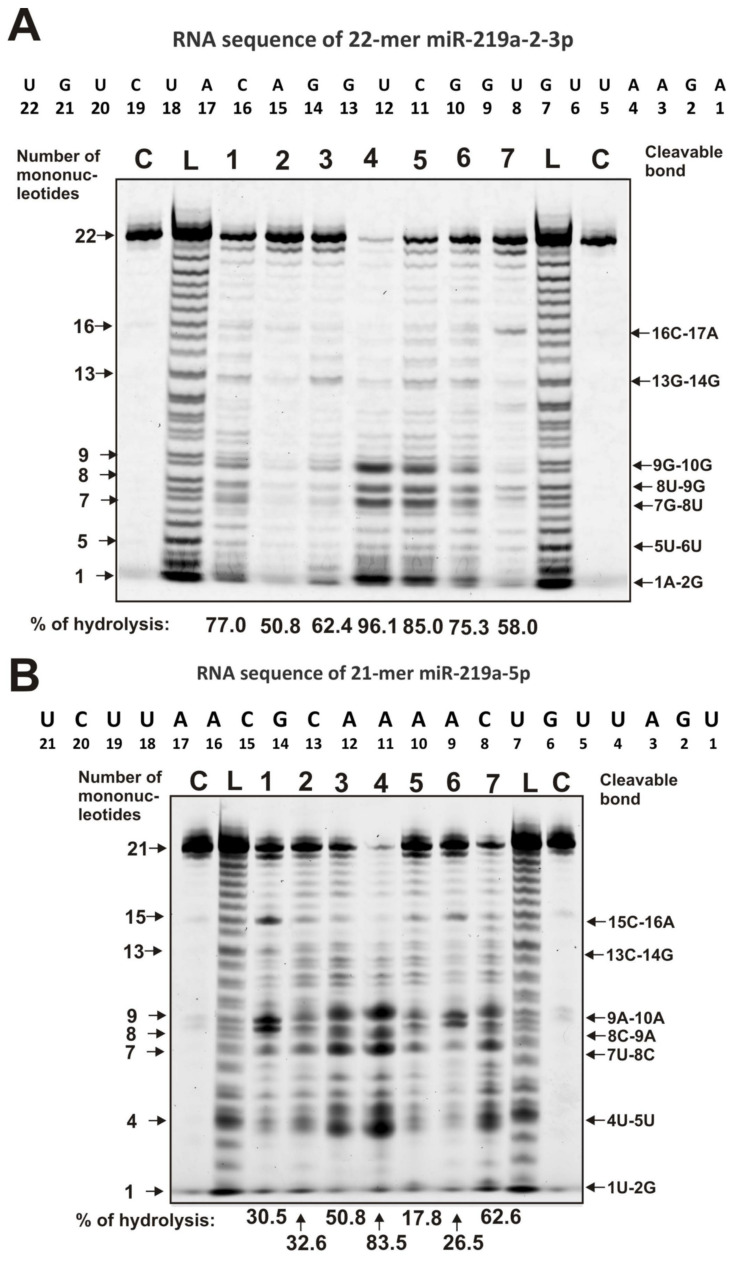
The patterns of 0.01 mg/mL F-miR-219a-2-3p (**A**) and F-miR-219a-5p (**B**) hydrolysis by seven human milk IgGs (lanes 1–7; 40 µg/mL). The products of miRNAs hydrolysis were detected due to their fluorescence (5′-Flu residues) after reaction mixture incubation for 1 h. The numbers of IgGs, the lengths of the oligonucleotide products, and the percentage of miRNAs hydrolysis by each IgG preparation are indicated in panels **A** and **B**. The L lanes correspond to the markers of oligonucleotide lengths, while C lanes correspond to the substrates incubated without IgGs. The percentages of hydrolysis correspond to the average values of three independent experiments.

In different mammals, some miRNAs regulate up to several hundred genes [55,56]. Specific splitting sites of miR-9-5p, miR-137, miR-219-2-3p, and miR-219a-5p were previously analyzed for IgGs from SCZ patients [63,64] and in the case of milk sIgAs [65]. These four miRNAs were also found in human milk [57,60]. MiR-219 participates in the differentiation of oligodendrocytes and axons of neuronal cell myelination [66]. Typical patterns of miR-219a-2-3p and miR-219a-5p hydrolysis by seven milk IgGs are given in Figure 3. The major cleavage sites of miR-219a-2-3p in the case of all seven IgGs are 9G-10G, 8U-9G, and 7G-8U, while there are four moderate sites of the splitting, i.e., 16C-17A and 13G-14G, 5U-6U, and 15C-16A (Figure 3A).

The products of miR-219a-5p cleavage by seven IgGs correspond mainly to five major sites of the hydrolysis: 9A-10A, 8C-9A, 7U-8C, 4U-5U, and 1U-2G (Figure 3B). All seven IgGs are less specific in the hydrolysis of miR-219a-5p than miR-219-2-3p. In contrast to miR-219a-5p, hydrolysis of miR-219a-5p, except five specific sites, is observed in almost all internucleoside phosphate groups of this miRNA; most of them can be attributed to average sites of the slitting (Figure 3B). Interestingly, IgG2 and IgG7 effectively hydrolyze three ribo-ONs nonspecifically at all sites (Figure 2), but, in contrast to miR-219-2-3p (Figure 3A), nearly the same situation for these two IgGs is observed in the hydrolysis of miR-219a-5p (Figure 3B). Interestingly, in the case of the predominant nonspecific splitting of miR-219a-5p, there are several typical major sites of this miRNA hydrolysis (Figure 3B).

**Figure 4:**
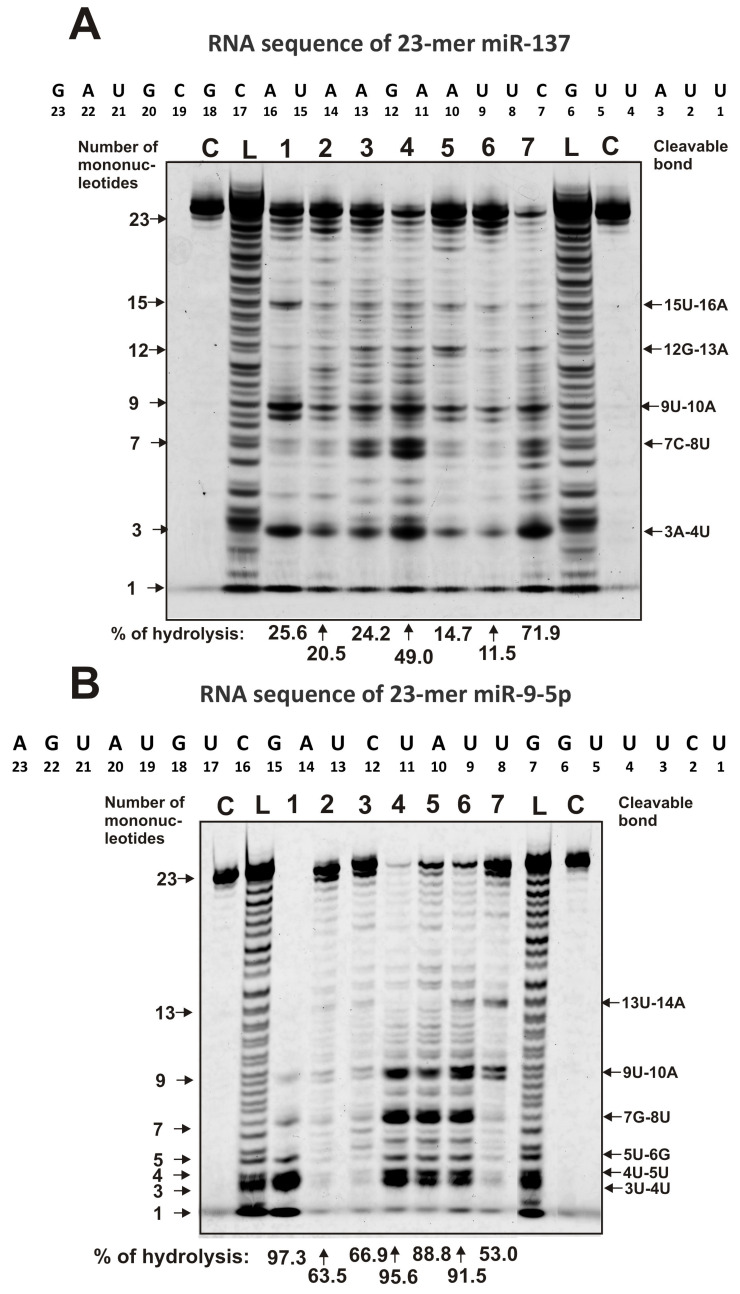
The patterns of 0.01 mg/mL Flu-miR-137 (**A**) and Flu-miR-9-5p (**B**) hydrolysis by seven human milk IgGs (lanes 1–7; 40 µg/mL). The products of miRNAs hydrolysis were detected due to their fluorescence (5′-Flu residues) after reaction mixtures incubation for 1 h. The numbers of IgGs, lengths of the oligonucleotide products, and the percentage of miRNAs hydrolysis by each IgG preparation are indicated in panels **A** and **B**. L lanes correspond to the markers of oligonucleotides lengths, while C lanes correspond to the substrates incubated without IgGs. The percentages of hydrolysis correspond to the average values of three independent experiments.

In the hydrolysis of miR-137, all IgGs demonstrate a combination of specific and site-specific hydrolysis of this miRNA. Among a large number of cleavage sites, including medium and weak ones, there are five major sites of hydrolysis, which are observed for all IgGs: 15U-16A, 12G-13A, 9U-10A, 7C-8U, and 3A-4U (Figure 4A). The effectiveness of the hydrolysis of mir-137 on major and other sites is very different for various IgGs.

All IgG preparations exhibit combined nonspecific and site-specific splitting of miR-9-5p (Figure 4B). Four preparations (IgG1, IgG4, IgG5, and IgG6) demonstrate strongly pronounced hydrolysis at four major sites: 9U-10A, 7G-8U, 4U-5A, and 3U-4U (Figure 4B). IgG6 and IgG7 show another 13U-14A major site of miR-9-5p hydrolysis. At the same time, for IgG7, the three main sites of hydrolysis (7G-8U, 4U-5A, and 3U-4U) are not major ones (Figure 4B). IgG2 and IgG3 hydrolyze this miRNA almost completely nonspecifically.

Altogether, all IgG preparations hydrolyze ribo-ONs nonspecifically, while in the case of four miRNAs, a combination of nonspecific and site-specific hydrolysis is observed. Thus, the patterns of hydrolysis of four miRNAs by each of the seven IgG preparations are individual, and some of them hydrolyze several miRNAs almost nonspecifically (Figure 3 and Figure 4).

The average RAs for different miRNAs and ribo-ONs were decreased in the following order (%): miR-9-5p (79.5 ± 17.9) ≥ miR-219a-2-3p (72.1 ± 15.9) > three ribo-ON (average value 59.0 ± 22.7) > miR219a-5p (43.7 ± 23.8) > miR-137 (31.2 ± 21.7). Thus, for milk IgGs, the best substrate is miR-9-5p, and the worst is miR-137.

Table 1 demonstrates the CCs between hydrolyzing activities of the corresponding seven different RNA-substrates. The CCs values are very different and vary from −0.55 [(pA)_23_ − miR-9-5p] to + 0.96 [(pA)_23_ − (pU)_23_]. The maximum negative correlation between four miRNAs was observed for miR-137 and miR-9-5p (CC = −0.43), while the maximum positive correlation was observed for miR-219-2-3p and miR-9-5p (CC = +0.86).

**Figure 5:**
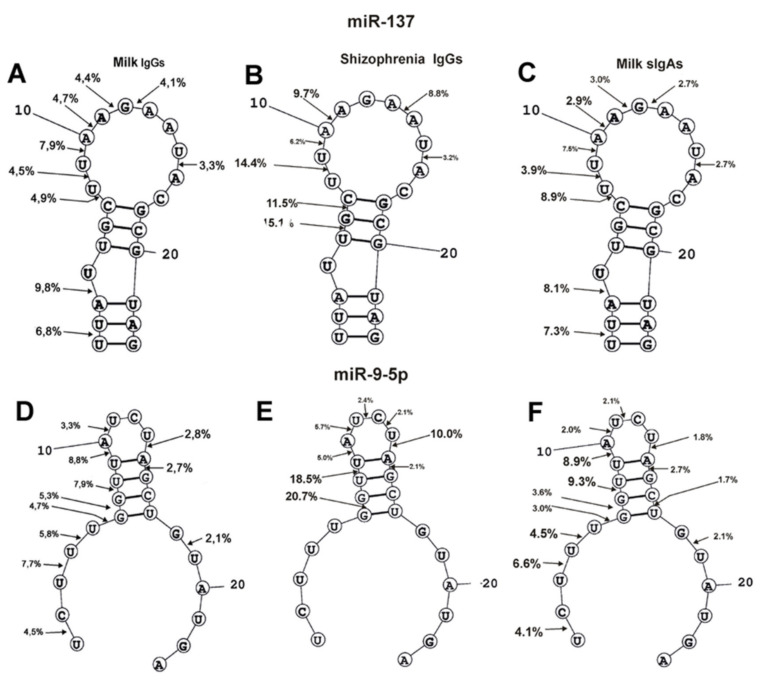
The average efficiency of F-miR-137 (**A–C**) and F-9-5p (**D–F**) by seven milk IgGs (**A**,**D**), IgGs of SCZ patients (**B**,**E**), and seven milk sIgAs (**C**,**F**) in all sites of their cleavage. The average percentage of two miRNA cleavages in different sites and the positions of major and moderate sites of miRNAs hydrolysis by all Abs are shown in the panels. For comparison, the data on the hydrolysis of miRNAs by milk sIgA [63] and the IgGs of schizophrenia patients [63,64] are taken from previously published articles.

**Figure 6:**
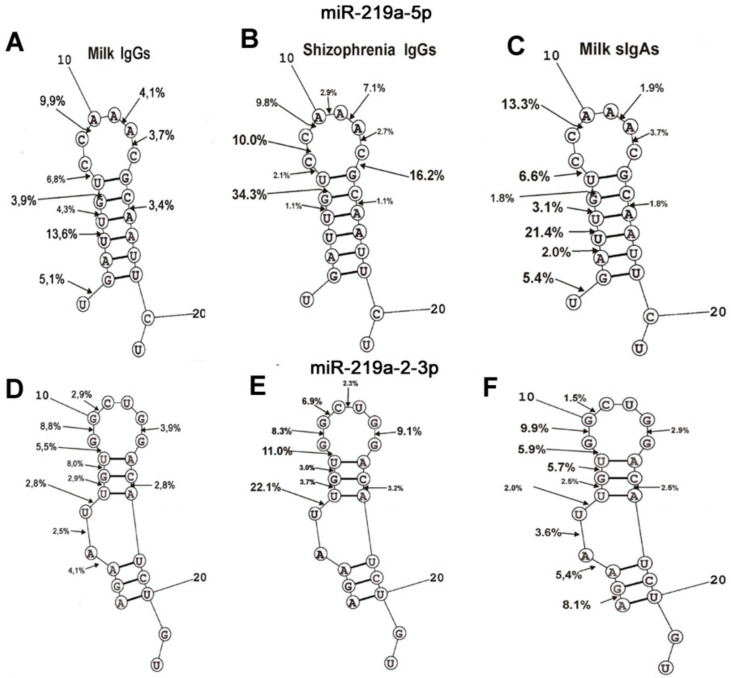
The average efficiency of Flu-miR-219a-5p (**A–C**) and Flu-miR-219a-2-3p (**D–F**) by seven milk IgGs (**A**,**D**), IgGs of SCZ patients (**B**,**E**), and seven milk sIgAs (**C**,**F**) in all sites of their cleavage. The average percentage of two miRNA cleavages at different sites and positions of major and moderate sites of miRNAs hydrolysis by all Abs are shown on the panels. For comparison, the data on the hydrolysis of miRNAs by milk sIgA [62] and the IgGs of schizophrenia patients [63,64] are taken from previously published articles.

The spatial structures of four miRNAs with minimal free energy were calculated earlier in [63,64]. The cleavage sites of four miRNAs from sera of SCZ patients were determined in [63,64]. In addition, there were determined site-specific splitting sites of these four miRNAs by sIgAs from human milk [65]. It was interesting to collate how much the four miRNAs cleavage sites coincide or differ in the case of milk IgGs, sIgAs, and IgGs of schizophrenia patients (Figure 5 and Figure 6). First, the relative amount (%) of every product of each miRNAs hydrolysis (from the intensity of all bands after electrophoresis) by individual IgGs was calculated. Then, based on the data of three independent experiments for each Abs sample, the average percentage of every product corresponding to seven milk IgG preparations was calculated (Figure 5 and Figure 6).

It should be noted that all nine sites of miR-137 hydrolysis are the same for milk IgGs and sIgAs (Figure 5). In the cleavage of miR-137 by SCZ IgGs, there is no noticeable hydrolysis of this miRNA in three of these nine specific sites (1U-2U, 3A-4U, and 7C-8U). However, SCZ IgGs split miR-137 in three other sites (5U-6G, 6G-7C, 13A-14A), in which hydrolysis by milk Abs is very weak (Figure 5). A significant difference in the hydrolysis of miR-137 lies in the fact that the major sites of its hydrolysis in schizophrenia IgGs are located in the middle of the molecule duplex part, while for milk Abs, they are located in the duplex part formed by the terminal fragments of this miRNA.

Ten cleavage sites of miR-9-5p by milk IgGs and sIgAs coincided completely (Figure 5B). Schizophrenia IgGs do not hydrolyze this miRNA in four sites of their 5′-terminal fragments (1U-2C, 3U-4U, 4U-5U, and 5U-6G) and at two sites of the 3′-terminal part of the molecule (16C-17U and 18G-19U), as well as at one site of miR-9-5p hairpin (7G-8U) (Figure 5). The hydrolysis of miR-9-5p by SCZ IgGs in its hairpin sequence occurs at three sites (8U-9U, 11U-12C, and 12C-13U), where there is a very weak cleavage of this miRNA by both milk antibodies. The main difference between the splitting of miR-9-5p by milk IgGs and sIgAs compared to schizophrenia IgGs is that the latter do not hydrolyze this miRNA at the 5′- and 3′-terminal sequences (Figure 5)

MiR-219a-5p cleavage sites for milk IgGs (9 sites) and sIgAs (10 sites) did not coincide completely; IgGs hydrolyze this miRNA very weakly at the 3A-4U site (Figure 6A). In the case of SCZ IgGs, 10 specific sites of cleavage were also found; however, several of them were different compared to those for milk IgGs and sIgAs. The cleavage sites characteristic of milk Abs (1U-2G, 3A-4U, and 4U-5U) are absent in the case of SCZ IgGs, but other distinctive sites specific for schizophrenia IgGs (8C-9C, 10A-11F, 13C-14G) were revealed. However, overall, the specific sites of miR-219a-5p hydrolysis by IgGs from the blood of patients with schizophrenia and both antibodies from milk were shown to be distributed throughout the entire miR-219a-5p molecule, except for its hydrolysis by milk IgGs and sIgAs at three sites (1U-2G, 3A-4U, and 4U-5U) of the 5′terminal sequence.

The number of hydrolysis sites of miR-219a-2-3p by milk IgGs (10 sites) and sIgAs (11 sites) was comparable, and the only sIgAs efficiently split it at 1A-2G 5′- terminal site (Figure 6B).

SCZ IgGs hydrolyzed miR-219a-2-3p at the same sites as milk IgGs except for three (1A-2G, 3A-4A, and 4A-5U) in the 5′-end of miRNA sequence.

Thus, milk IgGs and sIgAs hydrolyze four miRNAs, mostly at the same specific sites excluding several different ones (Figure 5 and Figure 6). However, the efficiency of the hydrolysis of four miRNA with milk IgGs and sIgAs at the same sites can be 1.5–2.5-fold different. Interestingly, much more significant differences are observed in the case of the hydrolysis of four miRNAs at the same specific sites by both milk and SCZ Abs. For example, the splitting of miR-219a-5p (6G-7G), miR-9-5p (6G-7G), miR219a-2-3p (5U-6U) at these sites by SCZ IgGs occurs respectively 8.8–19, 7.9-11.1, and 3.9–5.8 times more effectively than with milk IgGs and sIgAs (Figure 5 and Figure 6). In the case of other identical specific cleavage sites for SCZ IgGs and both milk Abs, significant differences are also observed. This may be due to the fact that in the case of SCZ IgGs, miRNA hydrolysis occurs more specifically without the formation of a large number of products of nonspecific hydrolysis compared with milk IgGs and sIgAs (Figure 5 and Figure 6). Given the presence of a large number of sites of weak nonspecific miRNAs hydrolysis, the relative percentage of their hydrolysis at specific sites is underestimated.

Seven sIgAs described earlier [65], and seven IgGs preparations analyzed in this work were obtained from the same milk samples. Therefore, it was interesting to estimate the CCs between the RAs of sIgAs and IgGs in the hydrolysis of three ribo-ONs and four miRNAs. We obtained unexpected results, i.e., the correlation coefficients between the RAs in the hydrolysis of the same substrates by milk IgGs and sIgAs were mainly negative: (pU) _23_(−0.85), (pA)_23_ (−0.7), miR219a-5p (−0.21), (pC)_23_ (−0.14), miR-137 (−0.09), except for the CCs for miR9-5p (+0.44) and miR-219a-2-3p (+0.26), which were positive.

## 3. Discussion

IgGs and IgAs from the blood of healthy donors, with very few exceptions, cannot usually hydrolyze RNA and DNA [67,68,69]. Milk IgGs and sIgAs of healthy lactating mothers effectively hydrolyze DNAs and polymeric RNAs [29,40].

In this article, we first showed that mother’s milk IgGs split different ribo-ONs and four miRNAs. The RAs in the hydrolysis of these RNA-substrates are highly dependent on the IgG preparations. Similar to SCZ IgGs [63,64] and milk sIgAs [65], milk IgGs hydrolyze ribo-ONs almost nonspecifically, thereby forming many products of different length (Figure 2). The correlation coefficients of the RAs between (pA)_23_ and (pU)_23_ was positive and high (+0.96), while CCs were negative for (pA)_23_ and (pC)_23_ (−0.61) and for (pU)_23_ and (pC)_23_ (−0.42). The average RAs in the hydrolysis of ribo-ONs are comparable (average value for three ONs = 55.0 ± 22.7%) and a little less than those for milk sIgAs (68.7 ± 23.7%) [65]. Interestingly, CCs between RAs in the hydrolysis of the same substrates by milk, sIgAs [65] and IgGs were mainly negative: (pU)_23_ (−0.85), (pA)_23_ (−0.7), miR219a-5p (−0.21), (pC)_23_ (−0.14), miR-137 (−0.09), except for miR9-5p (+0.44) and miR-219a-2-3p (+0.26), which were positive. This distinction may be due, to some extent, to the different origins of milk sIgAs and IgGs. The source of milk IgGs is still debated; they may be partially synthesized locally by specific cells of the female’s mammary gland and partially transferred from the mother’s blood circulation system [27]. IgAs are produced by B-lymphocytes of the local immune system of the mammary gland [28], and they are present in the mother’s Payer’s patch lymphoid cells, which migrate to mucosal sites and generate local secretory sIgA from two IgA molecules.

Using the Mann-Whitney test, there was no statistically significant difference between the hydrolysis of four microRNAs by sIgA; *P* value was higher than 0.05 [65]. A similar situation was observed for milk IgGs except for three pairs of RA sets: miR-137 and miR-219a-2-3p, miR-137 and miR9-5p, and miR219a-5p and miR9-5p (*p* ≤ 0.01).

Homologous ribo-ON are absolutely not homologous, but CCs in the hydrolysis of (pA)_23_ and (pU)_23_ are +0.96, while for (pA)_23_ and (pC)_23_, as well as for (pC)_23_ and (pU)_23_, are equal to −0.61 and −0.42, respectively. The homology of the four miRNAs is comparable (52–70.6%). Correlation coefficients in the hydrolysis of four miRNAs by milk IgGs are also not consistent with the level of their homology (CC-% homology): miR-137 – miR-9-5p (−0.43 and 70.6); miR-137-miR-219-2-3p (−0.07 and 52.0); miR-137-miR-219a-5p (+0.78 and 56.5); miR-9-5p-miR-219a-5p (−0.17 and 57.9); miR-9-5p-miR-219-2-3p (+0.86 and 59.1), and miR-219a-5p-miR-219-2-3p (+0.2 and 69.6).

For different miRNAs, several of the same major and moderate splitting sites were found in the case of milk IgGs, sIgAs [65], and SCZ [64] IgGs: miR-219-5p (U-G, G-U, C-A, A-A, and A-C); miR-219-2-3p (U-U, U-G, G-U, G-G, G-C, and C-A); miR-137 (U-U, U-A, and A-A); miR-9-5p (G-G, U-A, A-U, A-C) (Figure 5 and Figure 6). Thus, it turns out that miRNA cleavage sites can occur in any pair of any dinucleotides of their sequences. Therefore, it cannot be excluded that the nucleotide sequences of linear miRNAs do not play a substantial role in site-specific hydrolysis. It is possible that specific spatial conformations of miRNAs are more important for the position of the specific cleavage sites.

The specific cleavage sites of four miRNA hydrolysis by SCZ IgGs [60,61] and milk sIgAs [62], as well as the spatial structures of these RNAs, were found earlier. The spatial structures of miRNAs are given in Figure 5 and Figure 6. The positions and number of specific sites of miR-219-5p splitting with milk IgGs and sIgAs are nearly the same (Figure 6A). Interestingly, SCZ IgGs do not hydrolyze this miRNA at the 5′-dinucleotides of its duplex part (Figure 6A). MiR-219-2-3p has two duplex parts; the first one in the middle of the molecule, and the second is formed by terminal fragments of its sequence (Figure 6B). In spite of some differences in the specific cleavage sites of this miRNA in the first duplex part and hairpin structure, its hydrolysis by milk IgGs and sIgAs, as well as SCZ IgGs, is, to some extent, similar. The main difference is that, in contrast to SCZ IgGs, dairy IgGs and sIgAs hydrolyze the sites of 5′-terminal fragments of the second duplex part of miR-219-2-3p.

A very similar situation is observed in miR137 hydrolysis by both antibodies of human milk and IgGs from the blood of SCZ patients. The sites of the specific hydrolysis of the sequence of the hairpin structure of miR137 by SCZ IgGs and by both milk Abs almost coincide with each other (Figure 5A). The main difference is that SCZ IgGs hydrolyze this miRNA only in the first, while milk IgGs and sIgAs hydrolyze only in the second duplex part formed by terminal nucleotides of the molecule.

Figure 5B demonstrates that with a small difference in specific cleavage sites, SCZ IgGs, milk IgGs, and sIgAs hydrolyze the sequence of the hairpin structure of miR-9-5p somewhat similarly. MiR-9-5p does not form the second terminal duplex structure by its 5′- and 3′-sequences (Figure 5B). In contrast to SCZ IgGs, only milk IgGs and sIgAs demonstrate four specific cleavage sites in miR-9-5p 5′-terminal sequences. Apparently, milk IgGs and sIgAs can hydrolyze at specific sites of the 5′-terminal parts of the miRNAs, regardless of whether the duplex formed by terminal fragments of miRNAs exists.

Human milk and blood contain antibodies against both double- and single-stranded DNAs. Free DNA and RNA are very weak antigens [5,6,7,8,9]. Antibodies against nucleic acids are effectively produced when they are in complexes with any proteins. It is possible that, depending on different protein-forming complexes, the miRNAs in the blood of patients with schizophrenia, as well as in mother’s milk, mammary glands, and Payer’s patch, abzymes differing in specific sites of miRNA hydrolysis may be produced.

## 4. Materials and Methods

### 4.1. Chemicals and Donors

Most chemicals used were provided by Sigma (St. Louis, MO, USA). Protein G-Sepharose and Superdex 200 HR 10/30 columns were obtained from GE Healthcare (GE Healthcare, New York, NY, USA). Fluorescein isothiocyanate (FITC) was from Thermo Fisher (New York, MA, USA). FITC-conjugates of homogeneous ribooligonucleotides (ribo-ONs) were synthesized as in [70]. RNase A and thermosensitive alkaline phosphatase (FastAP) were bought from Fisher Scientific (Pittsburgh, PA, USA).

The milk sampling protocol was approved by the human ethics committee of State Medical University (Novosibirsk, Russia) in accordance with Helsinki ethics committee guidelines. All females gave written permission to use their milk for scientific purposes. The mothers had no history of any system pathologies.

### 4.2. Purification and Analysis of Antibodies

IgGs were purified from individual milk samples from seven healthy mothers (19–35 years old) from the Novosibirsk region (Russia) according to [29,49,50,51,52]. First, 100 mL was collected using standard sterile special devices at 1–3 weeks after the beginning of lactation. After 1–3 h of milk collection, all samples were cooled to 4 °C and centrifuged for 20 min at 14,000 rpm. Lipid phases and cells were removed to get milk plasma.

The plasma samples were loaded on a column with Protein G-Sepharose equilibrated by buffer A (Tris-HCl buffer (20 mM; pH 7.5), 0.15 M NaCl). Nonspecifically-bound proteins were first eluted from the column up to zero optical density using buffer A, and then with this buffer, were supplemented with 1% Triton X-100 and 300 mM NaCl, and again with buffer A. IgGs were eluted specifically from the column with 0.1 M glycine-HCl buffer (pH 2.6) destroying immunocomplexes, neutralized immediately using Tris-HCl (1 M; pH 8.5), and dialyzed using 20 mM Tris-HCl (pH 7.5).

For additional purification, IgG samples (0.3 mL; 1–5 mg/mL) were incubated in acidic glycine-HCl buffer (20 mM; pH 2.6) supplemented with 0.2 M NaCl at 20 °C for 20–30 min and subjected to FPLC gel filtration according to [29,42,43,44,45,46,47,48,49] on Superdex 200 HR 10/30. The fractions obtained were immediately neutralized by adding Tris-HCl (1 M, pH 9.0) and dialyzed as mentioned above. To refold the auto-Abs-ABZs after acidic treatment, their ribonuclease activity was estimated after storage for 1–2 weeks at 4 °C.

An immunoblotting analysis of the IgG_mix_ preparation was performed by Western blotting according to the standard procedure [71]. After SDS-PAGE, IgG_mix_ was transferred onto a nitrocellulose membrane. The membrane was treated with monoclonal mouse Abs (conjugated with horseradish peroxidase) against human IgGs or ribonuclease A.

### 4.3. Analysis of microRNA Hydrolysis by IgGs

Homogeneous 5’-F-(pA)_23_, 5’-F-(pU)_23_, 5’-F-(pC)_23_, and four miRNAs: miR-9-5p (5’-F-UCUUUGGUUAUCUAGCUGUAUGA), miR-219-2-3p (5’-F-AGAAUUGUGGCUGGACAUCUGU), miR-137 (5’-F-UUAUUGCUUAAGAAUACGCGUAG), and miR-219a-5p (5’-F-UGAUUGUCCAAACGCAAUUCU) contain fluorescent residue (fluorescein, F) on their 5’-termini. These four miRNAs were selected for the study since they are most commonly detected in human milk [56,57,60].

The reaction mixtures (10–15 μL) containing 50 mM Tris-HCl buffer (pH 7.5), 0.01 mg/mL of ribo-ON or miRNA, and 40 µg/mL IgGs were incubated at 37 °C for 1 h. A buffer (10 μL) containing 8 M urea and 0.025% xylene cyanol was added to the mixture to stop the reaction. The products of ribo-ON and miRNAs hydrolysis were revealed by 20% PAGE in a denaturing buffer (pH 8.3) containing 0.1 Tris, 0.02 M Na_2_EDTA, 0.1 M boric acid, and 8 M urea. Markers of ribo-ON and miRNAs length were obtained by their limited alkaline statistical hydrolysis and by RNA splitting using unspecific alkaline RNase hydrolyzing RNAs with comparable efficiency at all internucleoside bonds, as well as by specific RNase T1. After alkaline hydrolysis of ribo-ONs and miRNAs, all products of different lengths contained noticeable amounts cyclic 3’-monophosphate; therefore, they possessed lower electrophoretic mobility, giving additional bands. They were incubated with thermally sensitive FastAP alkaline phosphatase. A Typhoon FLA 9500 laser scanner (GE Healthcare, New York, UY, USA) was used for gel analysis. The results are given as a mean ± S.D. of at least three independent experiments.

### 4.4. In Situ RNase Activity Assay

An SDS-PAGE analysis of IgG samples (central part of the IgG peak after gel filtration) for homogeneity was performed in a 4–18% gradient gel containing 0.1% SDS (Laemmli system), as in [49,50,51,52]. An SDS-PAGE in situ analysis of the ribonuclease activity of IgG_mix_ (14 μg; an equimolar mixture of seven individual Abs) was carried out in 4–18% of PAGE containing 40 μg/mL of polymeric yeast RNA added to the mixture before copolymerization. Before SDS-PAGE, the IgG_mix_ was incubated at room temperature for 10–20 min in Tris-HCl (20 mM; pH 7.5) containing 0.1% SDS. The gels after electrophoresis were washed to remove SDS for 1–2 h using 20 mM Tris-HCl (pH 7.5) containing 0.1% Triton X-100, then three times with the same buffer, and finally, five times with 20 mM Tris-HCl. The gels were then incubated for 50–60 h at 37 °C in the reaction buffer (40 mM Tris-HCl, pH 7.5). The obtained gels were divided into two parts. One part was stained using ethidium bromide and was recorded with a Molecular Imager PharosFX Plus System (Bio-Rad, Berkeley, CA, USA). In the gel fragments in which polymeric RNA was split, a uniformly fluorescent background of the gel contain dark spots corresponding to the absence of polymeric RNA was observed. The other part of the gel was treated with Coomassie R-250 to find possible positions of the proteins.

#### 4.4.1. Spatial Model of microRNAs

The spatial models of four miRNAs were obtained in [63,64] using the Predict a Secondary Structure server to predict the structure of RNAs with minimal energy: http://rna.urmc.rochester.edu/RNAstructureWeb/Servers/Predict1/Predict1.html.

#### 4.4.2. Statistical Analysis

The relative activities of the IgGs were calculated from a decrease in the intensity of the fluorescence of the initial miRNAs (or ribo-ONs) in comparison with those for the control experiments corresponding to the incubation of the substrates without Abs. The results were given as the mean ± S.D. of at least three independent experiments for every IgG sample. The correlation coefficients (CCs) between different parameters were estimated using Microsoft Excel-2000. The differences between the RAs of different sets corresponding to various RNAs and IgGs were estimated using the Mann–Whitney test (Statistica 10; Statistical Package, StatSoft. Inc., Palo Alto, CA, USA; http://www.statsoft.com/Products/STATISTICA-Features); the value *p* < 0.05 was considered statistically significant.

## 5. Conclusions

In summary, we have shown that human milk IgGs evince specific miRNAs-hydrolyzing activities. The abzyme activity increase in milk may only be one part of the overall restructuring process of the women’s immune system leading to the production of autoantibodies and abzymes. The sIgAs and IgGs of milk manifest several unique enzymatic activities, including the phosphorylation of lipids, oligosaccharides, and proteins. It is not yet clear what important biological roles these unique abzymes hydrolyzing miRNAs can play in infant protection from various harmful factors. However, it should be assumed that the appearance of such human milk abzymes is not accidental.

Currently, some women consider it unnecessary to feed their newborns with their milk, choosing rather to substitute it with special baby food. However, food mixtures do not contain the mother’s milk antibodies and other specific components which provide passive immunity to newborns. Therefore, the analysis of the biological function of milk components, including Abs and abzymes, seems to be very important. In contrast to milk sIgAs, IgGs can penetrate the newborn’s blood through the intestinal epithelium, leading to the protection of cells and tissues of infants.

## Figures and Tables

**Table 1:** Values of coefficient correlations between the RAs of seven IgGs in the hydrolysis of miRNAs and ribo-ONs.

Substrate	(pU)_23_	(pC)_23_	miR-219a-5p	miR-219-2-3p	miR-137	miR-9-5p
(pA)_23_	+0.96	−0.42	+0.75	−0.27	+0.49	−0.55
(pU)_23_		−0.42	+0.80	−0.03	+0.40	−0.35
(pC)_23_			−0.22	+0.88	−0.02	+0.81
**Substrate**	**miR-219-2-3p**		**miR-137**		**miR-9-5p**	
miR-219a-5p	+0.2		+0.78		−0.17	
miR-219-2-3p			−0.07		+0.86	
miR-137					−0.43	

## Abbreviations

Abs: antibodies; ABZs, abzymes or catalytic antibodies; AI, autoimmune; AIDs, autoimmune diseases; CC, coefficient correlation; EAE, experimental autoimmune encephalomyelitis; EDTA, ethylenediaminetetraacetic acid; FITC, fluorescein isothiocyanate; Flu, fluorescent label; IgG, immunoglobulin G; HSC, hematopoietic stem cells; kDa, kilo Daltons; MS, multiple sclerosis; PAGE, polyacrylamide gel electrophoresis; NMP, NDP, and NTP, nucleoside mono-, di-, and triphosphate, respectively; RA, relative activity; SCZ, schizophrenia, SDS, sodium dodecyl sulfate; sIgA, secretory immunoglobulin A; SLE, systemic lupus erythematosus
